# Identifying Populations with Elevated PFAS Exposure by Targeted Serum Sample Pooling

**DOI:** 10.1007/s12403-025-00712-5

**Published:** 2025-05-20

**Authors:** Sandra Nilsson, Jennifer Bräunig, Ava Mueller, Nis-Julius Sontag, Daman Langguth, Carl Kennedy, Peter Hobson, Kevin V. Thomas, Jochen F. Mueller, Leisa-Maree Toms

**Affiliations:** 1 Queensland Alliance for Environmental Health Sciences, The University of Queensland, Woolloongabba, QLD Australia; 2 Environment Protection Science Branch, NSW Department of Climate Change, Energy, the Environment and Water, Lidcombe, NSW Australia; 3 Sullivan Nicolaides Pathology, Bowen Hills, QLD Australia; 4 Faculty of Health, School of Public Health and Social Work, Queensland University of Technology, Kelvin Grove, QLD Australia

**Keywords:** PFAS, Contamination, Biological monitoring, Cross-sectional studies

## Abstract

Per- and polyfluoroalkyl substances (PFAS) are a focus of biomonitoring studies globally. In Australia, population PFAS serum concentrations have been determined since 2002 using pooled de-identified serum samples (*n* = 4800) collected from the population bi-annually, as a component of the Australian Human Biomonitoring (HBM) project. Locations of environmental PFAS contamination (‘PFAS hotspots’) are known to exist in Australia, but the extent of human exposure at many of these hotspots remains unknown. This study assesses if systematic use of geographical pooling of surplus pathology samples can be effective to identify if elevated PFAS exposure has occurred in populations residing near ‘PFAS hotspots’. De-identified surplus pathology serum samples (*n* > 1800) were obtained and pooled from postcodes near three ‘PFAS hotspots’. Samples were analyzed for PFAS and compared with the Australian HBM project. Selected PFAS were consistently higher compared to the Australian HBM project in pools from two of the three ‘PFAS hotspots’, suggesting that these populations may have experienced elevated exposure to PFAS. This study demonstrates that targeted serum pooling can be an effective tool for determining indications of population exposure to persistent chemicals in communities at risk. The technique has the potential to be used for rapid surveillance and as a trigger for further investigations of populations in proximity to sites with known contamination.

**Graphical Abstract:**

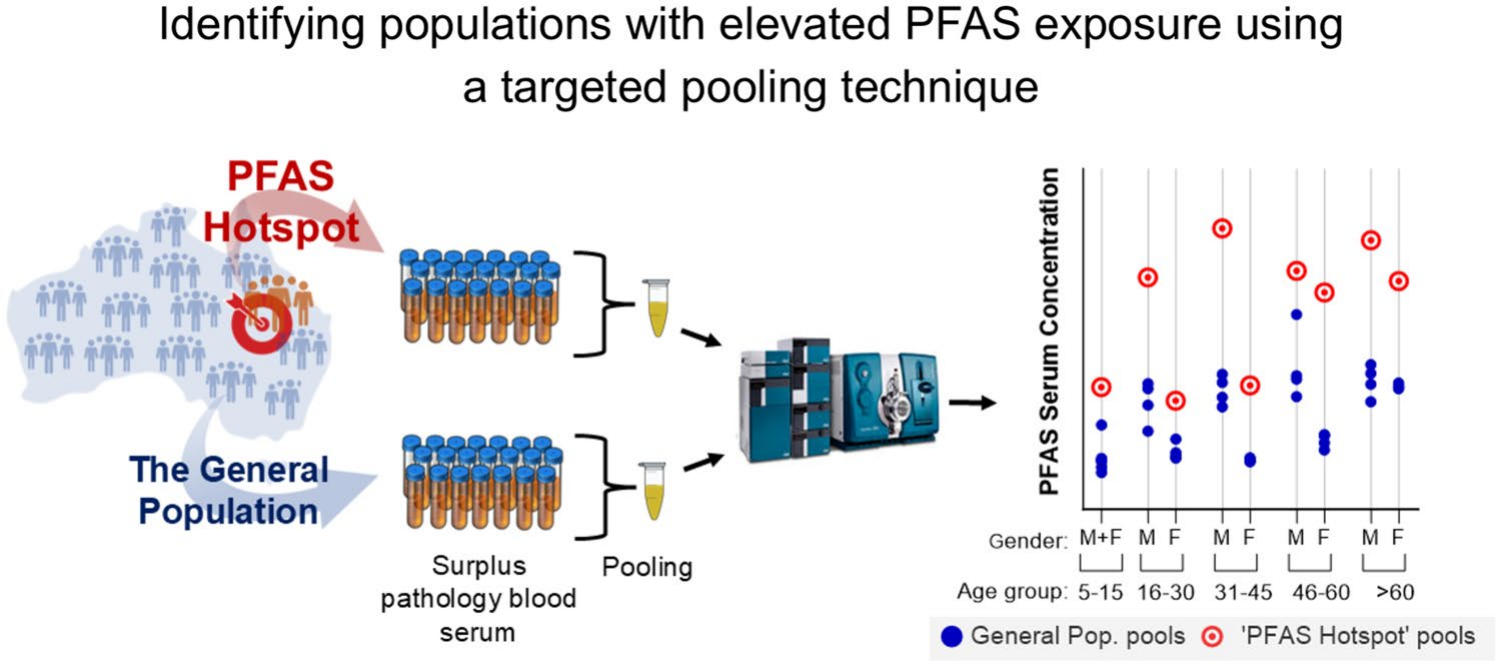

**Supplementary Information:**

The online version contains supplementary material available at 10.1007/s12403-025-00712-5.

## Introduction

Per- and polyfluoroalkyl substances (PFAS) are a group of synthetic chemicals that have been used extensively for more than 70 years as industrial additives and in a broad range of products, including aqueous film forming foams (AFFFs) (Key et al. 1997; Paul and Sweetman 2009; Prevedouros et al. 2006). PFAS have high resistance to thermal, chemical and biological degradation, and thus represent, or can break down to, very persistent compounds (Lau et al. 2007). Some of the most studied PFAS are perfluorooctanoic carboxylic acid (PFOA), perfluorohexane sulfonate (PFHxS) and perfluorooctane sulfonate (PFOS) which have human elimination half-lives commonly observed ranging from 3 to 8 years on average (Li et al. 2022a, b; Nilsson et al. 2022).

Exposure to PFAS represents a global health challenge and occurs predominantly through the food chain, contamination of water and through PFAS-containing consumer products (Sunderland et al. 2019). In addition to ‘background exposure’ sources, point sources include sites where PFAS-containing AFFF have been used and resulted in environmental contamination (‘PFAS hotspots’). Such sites may include airports, military bases, mining sites, refineries and other locations where large quantities of flammable or combustible materials have been stored (Hu et al. 2016). These contaminated sites have typically been identified when the site owner and/or the regulatory agency responsible for the environment/health commissioned environmental monitoring and PFAS was detected in water, soil, sediment, livestock and other media (Bräunig et al. 2017).

Determination of exposure risk for the local population is essential for adequate management at ‘PFAS hotspots’. For instance, the overall PFAS body burden of a potentially exposed population can provide an indication of whether the population has been exposed. Indeed, adequate risk assessment is needed to decide whether exposure reduction strategies are required, such as change of water source or water filtration (Committee on the Guidance on PFAS Testing and Health Outcomes. Guidance on PFAS Exposure 2022).

Environmental monitoring methods can be used to calculate the potential intake of PFAS via assessed pathways (e.g., water, soil, and air) and, in turn, to estimate human exposure and body burden. However, at the point in time when PFAS are detected through environmental monitoring, residents of the area may already have been exposed to this contamination for many years. An alternative method for assessing exposure and body burden in a population is human biomonitoring (HBM). Some of the most commonly used HBM techniques include random recruitment and sampling of people within a population cross-sectionally (e.g., the National Health And Nutrition Examination Survey (NHANES) in the USA (CDC 2021)), or longitudinal cohort studies where a group of individuals are recruited and sampled repeatedly over time.

In contrast to individual sampling, the ongoing ‘Australian HBM project’ uses pooled surplus pathology samples obtained cross-sectionally from the Australian population in a de-identified manner (by removing name, leaving only information regarding age, sex, postcode and sample collection date) (Toms et al. 2014). The advantages of this technique include the reduced cost of recruitment and sample analysis, as well as the speed at which sample collection and analysis can be completed to obtain a picture of the exposure in a certain population (Heffernan et al. 2014). The concentrations of PFAS in serum from the Australian population have been monitored as part of the Australian HBM project since 2002 and have provided evidence of decreasing PFAS exposure over this timespan (Taucare et al. 2024).

Various ‘PFAS hotspots’ of environmental contamination exist in Australia where PFAS has been reported in various media by monitoring programs or specific site investigations (e.g., pfas.australianmap.net 2025). At many of these sites, no comprehensive monitoring or exposure assessment has been conducted and thus, the level of human exposure is not known. Rapid identification of elevated human exposure at such point sources can enable governments and industry to react quickly to incidents or findings of environmental contamination.

The aim of this paper is to determine if the use of the established Australian HBM technique (i.e., cross-sectional pooling of surplus pathology samples), modified using targeted pooling protocols, can be used to investigate indications of elevated internal PFAS exposure in populations from ‘PFAS hotspots’ in Australia. To identify elevated exposure in communities at risk, up-to-date reference exposure data are essential. Therefore, the PFAS data from the two most recent Australian HBM project collections (2018/19 and 2020/21), which have not been published previously, are also presented in this paper.

## Materials and Methods

### Sample Collection

Ethics approval for this study was granted by The University of Queensland Medical Research Ethics Committee (2013000317) and Queensland University of Technology (1400000581). All samples were obtained in collaboration with Sullivan Nicolaides Pathology, a private pathology provider which is one of Australia’s largest laboratories for diagnostic testing. Samples were collected from individuals and analysed by Sullivan Nicolaides Pathology to assess certain biochemical parameters during medical diagnosis and therapy, and any ‘leftover’ serum remaining after these assessments (i.e., surplus pathology samples) were de-identified and made available for research purposes. Descriptive information available for each de-identified specimen was age, sex, date of collection and postcode. Individually unique codes allowed for no individual to have multiple samples included within or across pools. Selection of individuals was random without any exclusion criteria and only de-identified data (age, sex and postcode) of each sample included in the pool was recorded for each pool. Equal volumes from each sample were used and aliquoted into pools. The pooled samples were stored at − 20 °C until analysis.

### Pooling Protocol: Australian HBM Project

There were two collections of serum samples for the Australian HBM project conducted at the time of this study, in 2018/19 and in 2020/21. The pooling protocol was conducted according to previous collection rounds which has been described by Toms et al. 2009, 2014, 2019a, b. Samples were pooled according to sex (male, female) and age group: 0–4, 5–15, 16–30, 31–45, 46–60, and ≥ 60 years of age. Both age and sex are known factors associated with PFAS serum concentration (Sunderland et al. 2019). Four pools, consisting of 100 individuals, were constructed for each stratum (Table 1). This covers 4800 individual samples in each bi-annual sampling round.

**Table 1:** Details of total number of pools (individual samples/pool) collected for the Australian HBM project and the three site investigations (‘PFAS hotspots’)

Project		Location details	Pooling strategy								
			Postcodes	Gender	Age groups						Total individual samples
					0–4	5–15	16–30	31–45	46–60	> 60	
Australian HBM project	2018/2019	Nationwide	Nationwide postcodes	M	4 pools (*n* = 100)	4 pools (*n* = 100)	4 pools (*n* = 100)	4 pools (*n* = 100)	4 pools (*n* = 100)	4 pools (*n* = 100)	4800
				F	4 pools (*n* = 100)	4 pools (*n* = 100)	4 pools (*n* = 100)	4 pools (*n* = 100)	4 pools (*n* = 100)	4 pools (*n* = 100)	
	2020/2021	Nationwide	Nationwide postcodes	M	4 pools (*n* = 100)	4 pools (*n* = 100)	4 pools (*n* = 100)	4 pools (*n* = 100)	4 pools (*n* = 100)	4 pools (*n* = 100)	4800
				F	4 pools (*n* = 100)	4 pools (*n* = 100)	4 pools (*n* = 100)	4 pools (*n* = 100)	4 pools (*n* = 100)	4 pools (*n* = 100)	
Site Investigation	Site 1	Rural Australian town	One postcode covering entire town close to potential source	M	-	1 pool (*n* = 15)	1 pool (*n* = 88)	1 pool (*n* = 62)	1 pool (*n* = 45)	1 pool (*n* = 25)	582
				F	-		1 pool (*n* = 145)	1 pool (*n* = 98)	1 pool (*n* = 70)	1 pool (*n* = 34)	
	Site 2	Populous area of Australia, outskirts of major city	Postcodes within 5 km radius of potential source	M	20 pools (*n* = 10)*						920
				F	22 pools (*n* = 10)*						
			Postcodes within 10 km radius of potential source	M	25 pools (*n* = 10)*						
				F	25 pools (*n* = 10)*						
	Site 3	Populous area of Australia, outskirts of major city	Postcodes within 5 km radius of potential source	M	7 pools (*n* = 10)*						390
				F	9 pools (*n* = 10)*						
			Postcodes within 10 km radius of potential source	M	9 pools (*n* = 10)*						
				F	14 pools (*n* = 10)*						

^*^Pools from site 2 and 3 compromised of all age groups

Average age ranged from 28 to 60

### Pooling Protocol: ‘PFAS Hotspots’

Three sites, based on postcodes near suspected or known environmental PFAS contamination due to past use of PFAS-containing AFFF (‘PFAS hotspots’), were investigated in this study. We do not have any information on when the use of AFFF at any of these sites ceased. The selection of these three ‘PFAS hotspots’ was based on the criteria of having distinct postcodes geographically relevant to the hotspots, and sufficient sample numbers for pooling. No human exposure assessment has been conducted at these sites previously, thus it was uncertain whether widespread human exposure to PFAS had occurred in the populations at these sites. For the purpose of discretion, the names of the three sites are not disclosed. The three different sites are referred to as site 1, site 2 and site 3.

Samples were pooled according to the postcode of participants’ addresses that was recorded during routine pathology sample collection. As only age and sex information were available for each sample in addition to postcode no additional criteria could be applied for the pooling of samples. As samples were not collected from participants specifically for this study the availability of samples varied at each site. Thus, the pooling protocols were adapted for each of the locations.

#### Site 1

Site 1 is located in rural Australia and the entire town in proximity to the potential exposure source falls within one postcode. The PFAS point source includes a power station where PFAS-containing AFFF was used in the past. Serum was pooled according to the same age groups as per the Australian HBM project, resulting in a total of 9 pools covering five age groups (Table 1). For the youngest age group (5–15 years), males and females were pooled together due to low sample availability (15 individuals). Older age groups are stratified by sex due to higher availability of individual samples. Each pool consists of as many individuals as possible, ranging from 15 to 145. The samples comprising the pools for site 1 were all collected in 2021. For this reason*,* Australian HBM project data from 2020/2021 were used as reference data.

#### Site 2 and 3

Site 2 and 3 are located in more populous areas of Australia, with multiple postcodes surrounding each of the PFAS point sources (airports where PFAS-containing AFFF has been used in the past). Thus, samples from postcodes within 5 km and 10 km of the point source were pooled separately for sites 2 and 3. Pools are stratified by sex, but due to limited availability of individual samples not by age (i.e., pools include individuals of all ages). Each pool consists of 10 individuals, and multiple pools were collected from each location (Table 1). In contrast to the pooling protocol used for site 1 (including as many samples as possible within each strata), the creation of multiple pools compromising an equal number of individuals surrounding sites 2 and 3 allowed additional assessment of variation within each location, as well as a comparison between the 5 km and 10 km distances from the PFAS hotspot without being influenced by potential bias associated with unequal number of individuals compromising the pools. The samples from site 2 and site 3 were collected between 2018–2019, therefore the Australian HBM project data from 2018/2019 were used as a reference.

### PFAS Analysis

All samples were analysed at the Queensland Alliance for Environmental Health Sciences, using previously established analytical protocols (Nilsson et al. 2021; Toms et al. 2019a, b). Briefly, serum samples (200–300 µL) were spiked with mass labelled PFAS internal standard mix, vortex mixed and sonicated in 750 µl acetonitrile, centrifuged, filtered and concentrated before they were spiked with recovery mix. Up to 43 PFAS were analysed using a high-performance liquid chromatograph (HPLC, Nexera, Shimadzu Corp., Kyoto, Japan), coupled to a tandem mass-spectrometer (SCIEX Tiple Quad 6500 + , Concord, Ontario, Canada). Reported concentrations are for linear isomers, except for PFOS where the total concentration of both linear and branched isomers is reported. Method detection limits (MDLs) were set as 3.14 times the standard deviation of seven spiked serum samples, according to EPA guidelines (40 CFR 136), ranged from 0.13 to 0.40 ng/mL for PFOA, PFHxS and PFOS.

The serum separating tubes used by Sullivan Nicolaides Pathology have been tested for PFAS by adding MilliQ and extracting the MilliQ after various lengths of storing. No PFAS contamination was detected. We could not control for any potential contamination from previous pathology testing on the serum samples at the pathology laboratory. The laboratory takes part in an independent quality assurance program for the PFAS analysed and has passed assessments during this study’s timeframe (2018–2021). Further quality control/quality assurance (QC/QA) included the extraction and analysis of blanks (Acetonitrile, MilliQ and calf serum), inter and intra batch duplicates and replicates and standard reference materials (SRM, NIST 1957) alongside each batch of samples. In addition, in-house reference samples, spiked prior and post extraction were used to assess matrix effect and native recovery. Further analytical details and QC/QA outcomes are presented in the Supplementary Material (Tables S1, S2).

### Data Analysis

As equal volume of serum was used for each donor added into the pool, the concentration of PFAS in each pool represents the arithmetic mean concentration of all individual’s serum forming that pool. Thus, when presenting and comparing central tendencies in this manuscript, the arithmetic mean is used. Measurements below MDL are replaced by MDL/√2 for distribution calculations and statistical testing. Statistical tests were performed using GraphPad Prism.

There are two main aims of this paper; assess if there is any indication on population exposure near PFAS hotspots, as well as presenting up-to date population PFAS serum concentrations estimated from the two most recent Australian HBM project collections (2018/19 and 2020/21). For the 2018/19 and 2020/21 Australian HBM project only PFAS detected in > 80% of samples were used for further analysis, where we assessed the correlation between PFAS serum concentration and age (average age) (Pearsons correlation), and sex trends within each age group using point-biserial Pearsons Correlation (Kornbrot 2014).

For the purpose of assessing population exposure near the PFAS hotspots, only PFOA, PFHxS and PFOS are assessed in this paper. These PFAS are known markers of PFAS-containing AFFF-exposure, which is the suspected contaminant at these locations (Ying Li et al. 2022a, b; Nilsson et al. 2022; Smurthwaite et al. 2021). To assess if the PFAS exposure at the sites were elevated compared to the background levels expected in the Australian population, the mean PFAS concentration at each PFAS hotspot were compared against the corresponding mean of the Australian HBM Project data visually, and by calculating the percent difference. Due to the nature of the data obtained (different pooling protocols with limited and varied number of pooled samples at each site compared to the Australian HBM project), any further statistical analysis was not appropriate for this comparison.

## Results

PFOA, PFHxS and PFOS were detected (> MDL) in all pools collected as part of the routine Australian HBM project, as well as the three ‘PFAS hotspot’ sites. These PFAS are also known markers of AFFF-exposure, which is the main source of contamination assessed in this study. Thus, these three PFAS are the focus in this paper. Additional PFAS data are presented in the Supplementary Material (Tables S3–S5).

### Background PFAS Concentrations in the Australian Population

In the 2018–2019 collection, PFOA serum concentrations ranged from 1.2 to 2.8 ng/mL, PFHxS serum concentrations ranged from 0.71 to 4.1 ng/mL, and PFOS serum concentrations ranged from 1.8 to 8.5 ng/mL (plus one outlier pool of 13 ng/mL). In the 2020–2021 collection, the serum concentration of PFOA, PFHxS and PFOS ranged from 1.1 to 2.2. ng/mL, 0.64 to 3.4 ng/mL and 1.8 to 7.5 ng/mL, respectively. In accordance with previous Australian collections (Toms et al. 2019a, b), as well as other studies, PFAS concentrations were generally higher in males compared to females among adults (pools consisting of individuals of menstruating ages (i.e. 16–30, 31–45, 46–60 years)). Positive trends between PFHxS and PFOS concentrations and increasing age group were observed, but this was not significant for PFOA. Statistical outcomes (p-value summary), age and sex-specific PFAS measurements, as well as the detection frequency and concentration of all detected PFAS are presented in the Supplementary Material (Table S3).

In both collection years, the four pools from each stratum (age group/sex) showed consistent PFOA concentrations, with an average coefficient of variation (CV) of 9% [CV range from 5–15% (2018–2019 collection) and 2–18% (2020–2021 collection)]. The variation within stratum were not as consistent for PFHxS and PFOS (CV ranging between 6–40% (2018–2019 collection) and 2–32% (2020–2021 collection) for PFHxS, and 5–34% (2018–2019 collection) and 6–67% (2020–2021 collection) for PFOS). The larger variation was mostly contributed to by a few pools with significantly higher PFHxS/PFOS concentrations than the other pools within the same age group/sex (Fig. S1, Supplementary Material).

The Australian HBM project PFAS data were used as reference for the ‘PFAS hotspot’ pools. For comparisons, one Australian HBM project pool with significantly higher PFOS/PFHxS concentrations (double) than the other pools in the same strata (outlier) was excluded (*n* = 1, Male 45–60, 2018–2019 collection). The outlier pool was likely influenced by one, or a few, individuals with higher levels, e.g., due to elevated occupational exposure, and thus do not reflect the assumed ‘background exposure’. A comparison without exclusion of the outlier is presented in the Supplementary Material, but did not change any of the findings presented in the manuscript.

### Site-Specific Investigations

#### Site 1; Pooling by One Postcode

At site 1, pools were made up according to the same age groups as the Australian HBM project. Apart from the pool representing individuals from 5 to 15 years, where both male and female samples (n-15 individuals) were mixed to make up one pool, all other age groups were stratified by sex, consisting of samples from 62 to 145 individuals per each pool. Similar to the trends observed in the Australian HBM project, there was a positive association between PFAS serum concentration and age, and the concentrations of females were lower compared to males in the 16–30 and 31–45 age groups.

**Fig. 1:**
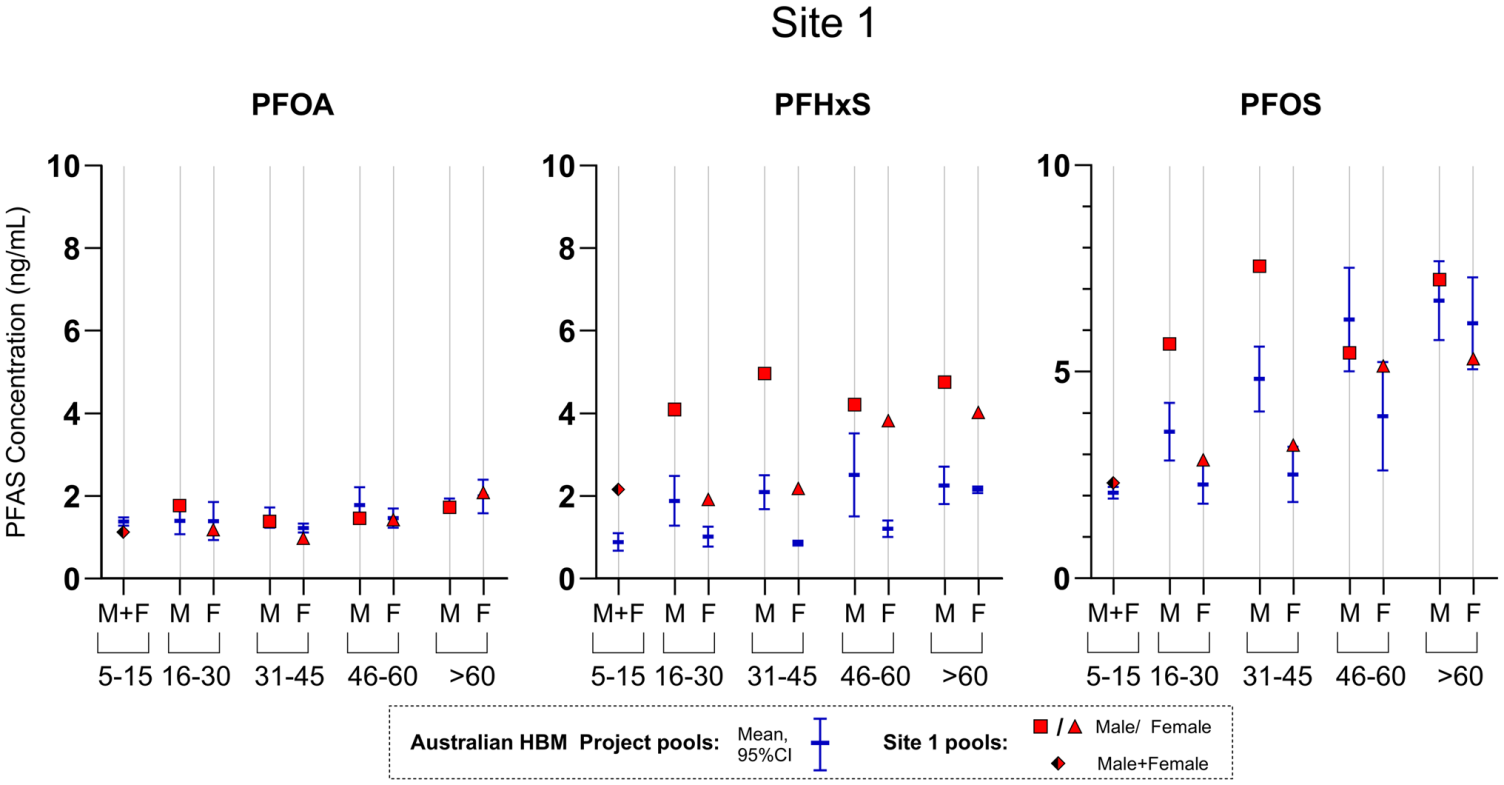
Concentrations of PFOA, PFHxS and PFOS in pools collected from site 1 in 2020 (red), and how they compare to respective concentrations measured in pools collected in the routine Australian HBM project in 2020–2021 (blue). Pools collected as part of the Australian HBM project were made up of 100 individuals/pool, where 4 pools were collected for each sex/age group. Pools collected from site 1 were made up of 15–145 individuals/pool

In Fig. 1. the concentrations of PFOA, PFHxS and PFOS measured in the pools from site 1 are compared to age and sex matched Australian HBM project pools. Overall, the PFOA concentrations from site 1 were similar, or slightly lower compared to the mean concentrations in the Australian HBM project pools. PFHxS concentrations were higher in pools from site 1 compared to the Australian HBM project pools across all age groups and sexes (ranging from 68 to 219% higher than the mean levels of the Australian HBM project). PFOS concentrations were higher compared to the Australian HBM project pool in adults (ages 16–45), most apparent in males. In the age groups 15–30 and 30–45, the pools from site 1 were 56–59% higher (males) and 25–28% higher (females), compared to the average Australian HBM project PFOS concentration. The PFOS concentrations in site 1 and the Australian HBM project pools were comparable in the younger (5–15 years) and older (> 60 years) age groups.

#### Site 2 and 3; Pooling According to Distance from the Source

**Fig. 2:**
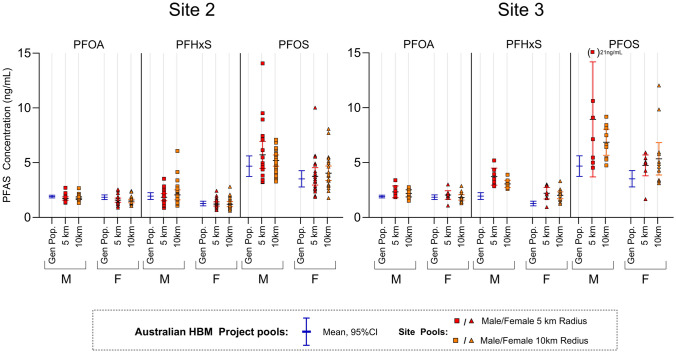
Concentrations of PFOA, PFHxS and PFOS in pools collected from site 2 and 3 in 2019, and how they compare to the mean Australian HBM project pools (blue) in 2018–2019. Pools collected as part of the Australian HBM project were made up of 100 individuals/pool, where 24 pools were collected for each sex. Pools collected from sites 2 and 3 were made up of 10 individuals/pools, where 20–25 pools were collected from each stratum at site 2, and 7–14 pools were collected from each stratum at site 3

At site 2 and 3, individuals living within a 5 km radius, and individuals living between 5–10 km radius from the potential ‘PFAS hotspot’ were pooled and referred to as the ‘5 km’ and ‘10 km’ pools from here on. Multiple pools, consisting of 10 individual samples/pool were collected. Pools were stratified by sex and include all ages. At both sites, PFAS serum concentrations were lower among females, compared to males. Mean (95% confidence interval) PFAS serum concentrations of the pools from site 2 and 3, compared to the mean Australian HBM pools are presented in Fig. 2.

At site 2, 92 pools were collected in total. No apparent difference in PFAS serum concentration was observed between the 5 km and 10 km radius pools. In both cases, the average PFAS concentration agreed with the average concentration of the Australian HBM pools (Fig. 2). It is worth noting that there was some variation in PFHxS and PFOS concentrations between pools, where maximum measured concentrations were approximately tenfold (PFHxS, 0.60 ng/mL-6.1 ng/mL) and eightfold (PFOS, 1.8 ng/mL-14 ng/mL) higher than in the pool with the lowest concentration, compared to PFOA where an approximate threefold difference is observed.

From site 3, 7–14 pools were collected for each radius and sex (29 in total). A slight trend can be observed when comparing the pools from the 5 km radius and 10 km radius in males, where the average PFHxS and PFOS concentrations in pools from the 5 km radius were 20% (PFHxS) and 30% (PFOS) higher compared to the 10 km radius pools. However, no such trend was apparent for females. Compared to the Australian HBM project, most pools from site 3 had higher PFHxS and PFOS concentrations. In male 5 km radius pools, average concentrations of PFHxS and PFOS were both approximately 90% higher than the Australian HBM pools concentration, and the 10 km radius pools were 47–57% higher compared to the Australian HBM pools. In female pools, average concentrations were 74% and 59% (PFHxS) and 35% and 52% (PFOS) higher in the 5 km and 10 km pools, respectively, compared to the Australian HBM pools. No apparent difference (< 11%) in mean PFOA concentrations between site 3 and the Australian HBM pools was observed for females. Mean PFOA concentrations were slightly (15–22%) higher than the male Australian HBM pools. Like site 2, there was a larger variation of measured PFHxS and PFOS concentration between pools, ranging from 0.94 ng/mL to 5.2 ng/mL for PFHxS, (5.5-fold difference) and 1.7 ng/mL to 21 ng/mL for PFOS (12-fold difference), compared to the variation of PFOA (threefold difference).

## Discussion

### PFAS Concentrations in the Australian Population

**Fig. 3:**
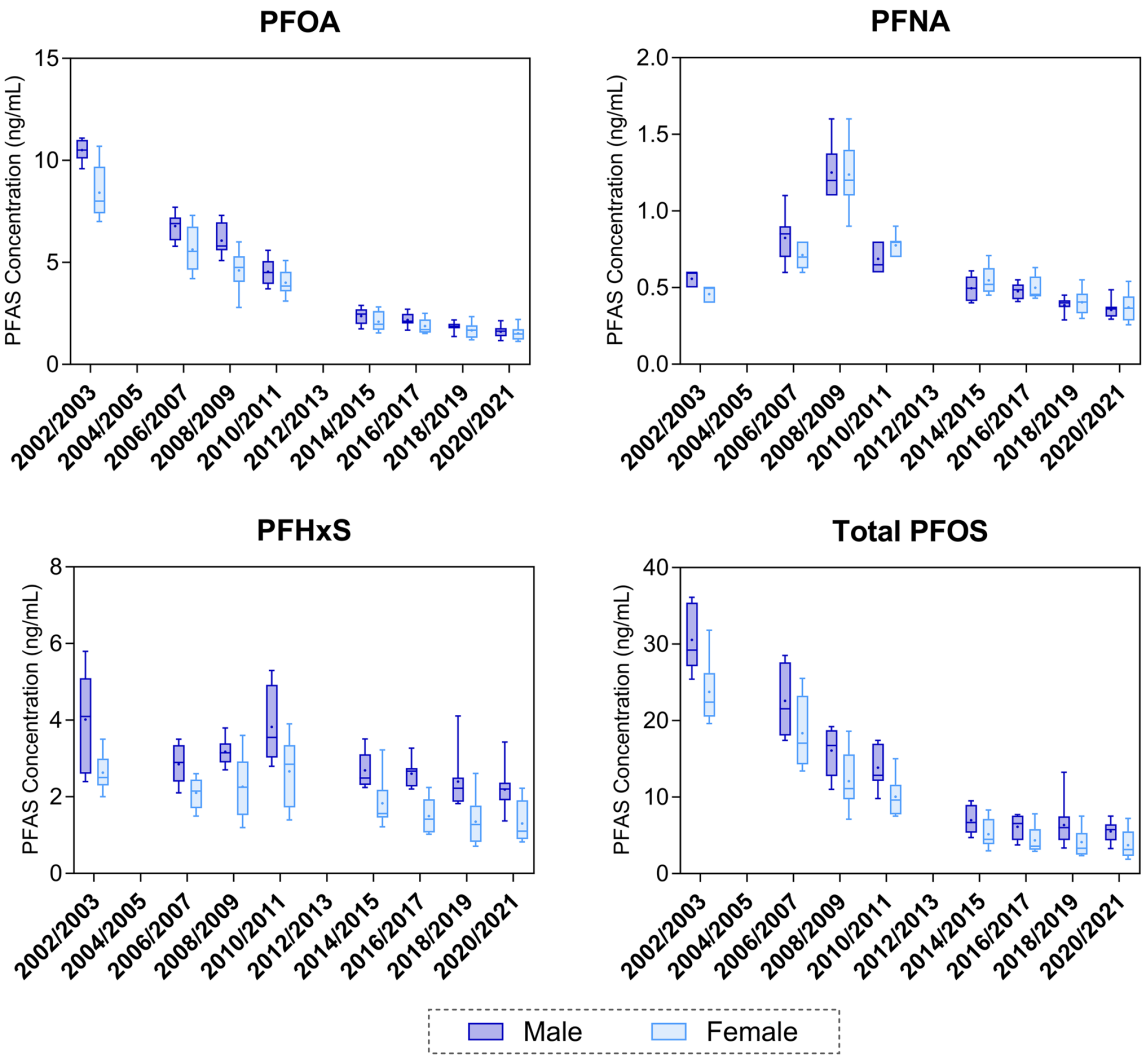
Temporal trend of PFAS concentrations (ng/mL) in serum pools of adults (> 16 years of age) collected from the Australian population (Australian HBM project). The 2018/2019 and 2020/21 bars represent the data presented in the current study. Bars showing previous collection rounds are obtained from previously published papers ( Toms et al. 2019a, b; Toms et al. 2014)

Bi-annually since 2002, the Australian HBM project has used pooled serum samples to monitor population exposures to PFAS ( Toms et al. 2019a, b; Toms et al. 2014). The two recent Australian HBM project collections in 2018/2019 and 2020/2021 provide further evidence of the decrease of the mean concentrations of the studied PFAS in serum from the Australian population, reflecting restrictions and limitations of PFAS that has been put in place in the last decades (Fig. 3) (Taucare et al. 2024). This decrease highlights the importance of up-to-date reference values when investigating whether measurements in a specific population are above typical levels to help identify communities or populations of concern.

Overall, low variation of PFOA concentrations between pools within each stratum (age group, sex, *n* = 4) suggest that the Australian population may be experiencing a relatively similar level of exposure to PFOA. The larger variation of PFHxS and PFOS concentrations may suggest a larger variance in exposure. Specific pools showed significantly higher concentration of PFOS or PFHxS compared to other pools within the same strata (Fig. S1). These pools have likely been influenced by one, or a few, individuals included in the pool that have experienced elevated exposure due to occupational exposure (e.g., firefighters) or environmental contamination.

### Identification of Populations with Elevated Exposure

By using targeted pooling of samples from specific geographical locations, we could identify indications of elevated PFHxS and PFOS exposure in pools from populations near ‘PFAS hotspots’. PFHxS and PFOS are major ingredients in certain AFFFs, which is the suspected source of contamination at the selected sites. Elevated levels of PFHxS and PFOS have previously been found in firefighters who have worked with PFAS-containing AFFF in the past, as well as individuals from three communities affected by environmental contamination (PFAS management sites) in Australia (Lazarevic et al. 2021; Nilsson et al. 2022).

At site 1, we found elevated concentration of PFHxS consistently in both male and female pools in all age groups, compared to the population pools. Elevated levels of PFOS were also apparent for males in specific age groups (15–30, 31–45), while this elevation was not clear in female pools.

In comparison to PFOS, PFHxS is more water-soluble and less proficient to adsorb to organic matter and thus more environmentally mobile (IPEN 2019; Nguyen et al. 2020). The more apparent elevation of PFHxS, compared to PFOS at site 1, may be an indication of a contamination plume where PFHxS is typically transported faster due to lower retention on solids/soils (IPEN 2019; Nguyen et al. 2020). Hence this may be an early sign for potential future exposure to additional PFAS. As elevated exposure to PFHxS was consistently apparent throughout all strata, there is the potential for this exposure to come from a common/widespread exposure source, such as a contaminated water supply. The study findings of this site were provided to relevant agencies to act upon at their own discretion. Follow up biomonitoring is currently being undertaken at this site.

At site 3, nearly all pools showed higher PFHxS and/or PFOS concentrations compared to the Australian HBM project. This consistent trend suggests that elevated exposure is widespread in the population at this site. The higher mean PFHxS/PFOS in closer proximity to the ‘PFAS hotspot’ support the suggestion that PFAS exposure is associated with the ‘PFAS hotspot’. At site 2, only some pools showed higher concentrations of PFHxS/and or PFOS compared to the Australian HBM project pools. The inconsistent elevation of PFHxS/PFOS concentration in pools, and the fact that the mean PFAS concentrations were consistent with the Australian HBM project, suggest that there is no widespread elevated exposure in the population at this site. This is also supported by the lack of trend in association with distance from the PFAS hotspot. It is possible that the pools with higher concentrations at this site are influenced by one, or a few individuals, that have experienced elevated exposure. Higher PFAS concentrations shown in just a few pools may be due to specific exposures (e.g., occupational exposure, consumption on local produce or use of bore water) rather than environmental exposure that has affected a water source (which would be expected to be more widespread in the population around a source). The large variation observed in PFHxS/PFOS concentration at Site 2 and 3 is noteworthy as specific exposure sources may be difficult to identify as part of environmental monitoring and further highlights the potential use of this method as a first screening tool.

### Pooling Strategy Considerations

Where an indication of elevation was observed (Site 1 and 2), the clearest elevation was observed in males, perhaps because of faster elimination rates in females (e.g., through menstruation, pregnancy and lactation (Gomis et al. 2017)). It is also possible this is influenced by potential occupational AFFF-exposure at these sites in male-dominated jobs (Nilsson et al. 2022). Case-specific pooling protocols will be dependent on sample availability, but where possible it should aim to reflect known population variability by sex and age stratification, as well as create multiple pools to assess any potential population variability.

Both at site 2 and 3, where multiple pools were collected from each strata, variance especially in PFHxS and PFOS concentrations was observed. Variation between pools is not unexpected due to the low number of individual samples included in each pool (*n* = 10), where individual samples with outlying concentrations have a greater influence on the mean. Furthermore, these pools were not stratified by age. In this study, we could not assess whether or not this variance was different to the variance in concentrations within the Australian background population. The Australian HBM project data used as reference consists of a larger number of individuals in each pool (*n* = 100 individuals per pool), and the variance between these pools was thus not comparable to the variance between pools collected from site 2 and 3. To identify any potential heterogenous population exposure at a specific site, multiple pools are necessary. However, to assess if the variance is different from the underlying population variance, the reference pooling protocol should be comparable.

When interpretating results of pooled samples, it is important to consider the expected difference that is expected between ‘PFAS hotspots’ and the reference population. Pools represent the arthritic mean, and thus, extreme PFAS concentrations are not expected unless the whole population is highly exposed. In the case of environmental contamination, unless a common drinking water source is impacted, exposure may arise from specific exposure routes (such as eating local produce or using bore water) which may only impact a small proportion of the population. For example, a recent study measuring PFAS serum concentrations in individuals from three AFFF-impacted communities in Australia found that 29%-42% (PFOS) to 48%–55% (PFHxS) of individuals had elevated serum concentrations compared to corresponding reference communities (Smurthwaite et al. 2021). Thus, a pooled sample from ‘PFAS hotspots’ may be highly influenced by inclusion of non-exposed individuals, underestimating the extent of exposure in specific individuals in the area. This is especially important to consider in the case of postcode specific pooling, as one postcode may cover both contaminated and non-contaminated areas. Of course, on the other hand inclusion of a few highly exposed individuals may overestimate the exposure in the population, especially in pools with small sample sizes, discussed further in the next section. Although it is not always possible when using samples of convenience, the number of samples included in each pool should ultimately be the same in both the site of interest and the reference, to minimise the abovementioned limitations of comparing arthritic means.

### Application of the Current HBM Technique

The HBM technique used in this study has been used to monitor population exposure in Australia since 2002 and has been effective for identifying both temporal and cross-sectional trends of PFAS exposure in the population. The pooling of samples can substantially reduce time and analytical costs of traditional human biomonitoring. Furthermore, using (surplus) samples that were initially collected for pathology testing, reduces time and resources required for recruitment. The burden experienced by study participants, such as physical and financial burdens as well as stress/anxiety, is also avoided. These advantages allow for a quick response when investigating exposure in communities of concern. For example, following the news about potential contamination at site 1, we could confirm/identify indications of elevated exposure in the community in a little under two weeks, which allowed a rapid governmental response. It is worth to note, that some small/remote sites may have additional time constrains while waiting for enough samples to be taken and made available for use.

Using this technique to investigate populations who may be at risk due to proximity to potential contamination sources provides a snapshot of community exposure and may help provide an early warning for a population. Reporting early findings of elevated exposure can enable the government to undertake assessment for further decision making in a timely manner. Monitoring the exposure in populations at risk can also be helpful for recommendations on consumption of local produce and exchange of water sources. Continuous monitoring of contaminated sites over time can confirm if exposure control attempts are effective in reducing exposure, as long as sufficient time is left between sampling points, to allow the change in PFAS serum concentration to be greater than the analytical uncertainty (Nilsson et al. 2021). In addition to assessing populations in proximity to environmental contamination, strategic postcode pooling can also be useful for understanding general exposure differences across geographical areas, which may be influenced by differences is socioeconomics, lifestyles, diets, or use of consumer products.

It is important to note the limitations of the HBM technique used in this study. Limitations include the potential biases using samples of convenience, i.e., samples collected for routine pathology tests from potentially sick people. Samples biased towards sick people may not be reflective of the general population at the site investigated, especially if the exposure is associated with health outcomes that requires pathology testing. Further, the use of de-identified samples means that the length of residence is not known (i.e., length of exposure). Nor are potential factors affecting exposure/PFAS serum concentrations known (e.g., occupational exposure, potential health issues such as decreased liver function). The pooling criteria was limited to postcode, age and sex, and due to the differences in sample availability at different sites, consistent pooling protocols are not always possible. Inconsistent pooling protocols, due to the use of samples of convenience can limit statistical comparisons to bolster the robustness of observations. The ‘Australian HBM project’, which was used as a proxy for ‘background exposure’ was collected independently from the ‘PFAS hotspot pools’. The Australian HBM project consist of randomly collected specimen to reflect overall exposure, with no exclusion criteria. In hindsight, we found that the 2018–2019 collection included specimens from some of the postcodes representing ‘the PFAS hotspots’. However, the inclusion of such postcodes was relatively consistent across strata and represented less than 2% of the specimens. It is unlikely that this would affect have influenced the results significantly. The Australian HBM project may also be influenced by individuals from currently unknown ‘PFAS hotspots’ or those that have experienced occupational exposure.

Furthermore, pooling of samples does not provide the true underlying variation within a population. As discussed above, the pooling of samples provides the ‘arthritic mean’, which may be skewed if one or a few individuals with abnormally high levels is included in the pool, especially in pools with low sample numbers, which may explain the large variation of PFHxS and PFOS among pools from site 2 and 3, compared to the Australian HBM project pools. The measured concentrations in the pools may also be influenced by non-exposed individuals if the exposure in the ‘PFAS hotspots’ are only affecting a small proportion of the population. Thus, it is important to consider that although the current technique may be able to identify indications of elevated exposure, it may not be able to determine with certainty that elevated exposure has not occurred if this elevated exposure is not widespread in the community. It is worth to note, that we have not conducted a comparative exposure assessment using individual samples at the ‘PFAS hotspots’ to confirm the trends observed. Nevertheless, this study shows that it is possible to detect an indication of exposure using pooled serum samples of convenience that can trigger further more comprehensive studies (such as individual testing) to confirm the exposure trends in these populations and help prioritise communities at risk.

Pooling of serum samples has been used for over two decades to determine exposure in the Australian population. This study demonstrates that the concept of serum pooling by a set variable (postcode, in this case) may also be an effective tool in determining population exposure in communities at risk. Comparative analysis using individual samples in such communities will be able to confirm this. While in the current study, we investigated the application of this concept for PFOA, PFHxS and PFOS exposure, this technique has potential application to other emerging contaminants of concern. The technique may be used for rapid surveillance of exposure as the limitations are outweighed by the ability to provide a cost-effective and timely response to community and/ or government concerns regarding geographical region-specific exposure to environmental contaminants.

## Supplementary Information

Below is the link to the electronic supplementary material.

Supplementary file1 (DOCX 219 KB)

## Data Availability

The authors confirm that the data supporting the findings of this study are available within the article and supplementary materials. Derived raw data supporting the findings of this study may be available in de-identified manner from the corresponding author on request.

## References

[CR1] Bräunig J, Baduel C, Heffernan A, Rotander A, Donaldson E, Mueller JF (2017) Fate and redistribution of perfluoroalkyl acids through AFFF-impacted groundwater. Sci Total Environ 596–597:360–368. 10.1016/j.scitotenv.2017.04.095 10.1016/j.scitotenv.2017.04.095 28441576

[CR2] CDC, C. f. D. C. (2021). About the national health and nutrition examination survey. https://www.cdc.gov/nchs/nhanes/about_nhanes.htm Accessed 16 Sept 2021

[CR3] Committee on the Guidance on PFAS Testing and Health Outcomes. Guidance on PFAS Exposure, T., Clinical Follow-Up. . (2022). Appendix E, white paper: review of the PFAS personal intervention literature. https://www.ncbi.nlm.nih.gov/books/NBK584698/

[CR4] Gomis MI, Vestergren R, MacLeod M, Mueller JF, Cousins IT (2017) Historical human exposure to perfluoroalkyl acids in the United States and Australia reconstructed from biomonitoring data using population-based pharmacokinetic modelling. Environ Int 108:92–102. 10.1016/j.envint.2017.08.002 28818713 10.1016/j.envint.2017.08.002

[CR5] Heffernan A, Aylward L, Toms L-M, Sly P, MacLeod M, Mueller JF (2014) Pooled biological specimens for human biomonitoring of environmental chemicals: opportunities and limitations. J Eposure Sci Environ Epidemiol. 10.1038/jes.2013.76 10.1038/jes.2013.76 24192659

[CR6] Hu XC, Andrews DQ, Lindstrom AB, Bruton TA, Schaider LA, Grandjean P, Lohmann R, Carignan CC, Blum A, Balan SA, Higgins CP, Sunderland EM (2016) Detection of poly- and perfluoroalkyl substances (PFASs) in U.S. drinking water linked to industrial sites, military fire training areas, and wastewater treatment plants. Environ Sci Technol Lett 3(10):344–350. 10.1021/acs.estlett.6b00260 27752509 10.1021/acs.estlett.6b00260 PMC5062567

[CR7] IPEN. (2019). Perfluorohexane sulfonate (PFHxS)—socio-economic impact, exposure, and the precautionary principle.

[CR8] Key BD, Howell RD, Criddle CS (1997) Fluorinated organics in the biosphere. Environ Sci Technol 31(9):2445–2454

[CR9] Kornbrot, D. (2014). Point biserial correlation. In *Wiley StatsRef: statistics reference *10.1002/9781118445112.stat06227

[CR10] Lau C, Anitole K, Hodes C, Lai D, Pfahles-Hutchens A, Seed J (2007) Perfluoroalkyl acids: a review of monitoring and toxicological findings. Toxicol Sci 99(2):366–394 17519394 10.1093/toxsci/kfm128

[CR11] Lazarevic, N., Smurthwaite, K., Trevenar, S., D’Este, C., Batterham, P., Lane, J., Armstrong, B., Lucas, R., Clements, A., Banwell, C., Hosking, R., Joshy, A., Gad, I., Law, H.-D., Mueller, J., Bräunig, J., Nilsson, S., Lal, A., Randall, D., Kirk, M. (2021). PFAS health study component three: cross-sectional survey of self-reported physical and mental health outcomes and associations with blood serum PFAS.

[CR12] Li Y, Andersson A, Xu Y, Pineda D, Nilsson CA, Lindh CH, Jakobsson K, Fletcher T (2022a) Determinants of serum half-lives for linear and branched perfluoroalkyl substances after long-term high exposure-a study in Ronneby. Sweden Environ Int 163:107198. 10.1016/j.envint.2022.107198 35447437 10.1016/j.envint.2022.107198

[CR13] Li Y, Andersson A, Xu Y, Pineda D, Nilsson CA, Lindh CH, Jakobsson K, Fletcher T (2022b) Determinants of serum half-lives for linear and branched perfluoroalkyl substances after long-term high exposure—a study in Ronneby. Sweden Environment International 163:107198. 10.1016/j.envint.2022.107198 35447437 10.1016/j.envint.2022.107198

[CR14] Nguyen TMH, Bräunig J, Thompson K, Thompson J, Kabiri S, Navarro DA, Kookana RS, Grimison C, Barnes CM, Higgins CP, McLaughlin MJ, Mueller JF (2020) Influences of chemical properties, soil properties, and solution pH on soil-water partitioning coefficients of per- and polyfluoroalkyl substances (PFASs). Environ Sci Technol 54(24):15883–15892. 10.1021/acs.est.0c05705 33249833 10.1021/acs.est.0c05705

[CR15] Nilsson S, Mueller JF, Rotander A, Bräunig J (2021) Analytical uncertainties in a longitudinal study—a case study assessing serum levels of per- and poly-fluoroalkyl substances (PFAS). Int J Hyg Environ Health 238:113860. 10.1016/j.ijheh.2021.113860 34649073 10.1016/j.ijheh.2021.113860

[CR16] Nilsson S, Smurthwaite K, Aylward LL, Kay M, Toms LM, King L, Marrington S, Barnes C, Kirk MD, Mueller JF, Bräunig J (2022) Serum concentration trends and apparent half-lives of per- and polyfluoroalkyl substances (PFAS) in Australian firefighters. Int J Hygiene Environ Health 246:114040. 10.1016/j.ijheh.2022.114040 10.1016/j.ijheh.2022.114040 36162311

[CR17] Paul AGJKC, Sweetman AJ (2009) A first global production, emission, and environmental inventory for perfluorooctane sulfonate. Environ Sci Technol 43(2):386–392 19238969 10.1021/es802216n

[CR18] pfas.australianmap.net. (2025). The Australian PFAS chemicals map. https://pfas.australianmap.net/

[CR19] Prevedouros K, Cousins IT, Buck RC, Korzeniowski SH (2006) Sources, fate and transport of perfluorocarboxylates. Environ Sci Technol 40:32–44 16433330 10.1021/es0512475

[CR20] Smurthwaite, K., Lazarevic, N., Bräunig, J., Mueller, J., Nilsson, S., D’Este, C., Lucas, R., Armstrong, B., Lal, A., Trevenar, S., Law, H.-D., Gad, I., Hosking, R., Joshy, A., Clements, A., Lane, J., Batterham, P., Banwell, C., Miller, A., Kirk, M. (2021). PFAS Health study component two: blood serum study of PFAS exposure, related risk factors and biochemical markers of health.

[CR21] Sunderland EM, Hu XC, Dassuncao C, Tokranov AK, Wagner CC, Allen JG (2019) A review of the pathways of human exposure to poly- and perfluoroalkyl substances (PFASs) and present understanding of health effects. J Eposure Sci Environ Epidemiol 29(2):131–147. 10.1038/s41370-018-0094-1 10.1038/s41370-018-0094-1 PMC6380916 30470793

[CR22] Taucare G, Chan G, Nilsson S, Toms L-ML, Zhang X, Mueller JF, Jolliet O (2024) Temporal trends of per- and polyfluoroalkyl substances concentrations: insights from Australian human biomonitoring 2002–2021 and the US NHANES programs 2003–2018. Environ Res 262:119777. 10.1016/j.envres.2024.119777 39155039 10.1016/j.envres.2024.119777

[CR23] Toms L-ML, Calafat AM, Kato K, Thompson J, Harden F, Hobson P, Sjödin A, Mueller JF (2009) Polyfluoroalkyl chemicals in pooled blood serum from infants, children, and adults in Australia. Environ Sci Technol 43(11):4194–4199. 10.1021/es900272u 19569351 10.1021/es900272u

[CR24] Toms LM, Thompson J, Rotander A, Hobson P, Calafat AM, Kato K, Ye X, Broomhall S, Harden F, Mueller JF (2014) Decline in perfluorooctane sulfonate and perfluorooctanoate serum concentrations in an Australian population from 2002 to 2011. Environ Int 71:74–80. 10.1016/j.envint.2014.05.019 24980755 10.1016/j.envint.2014.05.019 PMC4724209

[CR25] Toms LM, Bräunig J, Vijayasarathy S, Phillips S, Hobson P, Aylward LL, Kirk MD, Mueller JF (2019a) Per- and polyfluoroalkyl substances (PFAS) in Australia: current levels and estimated population reference values for selected compounds. Int J Hyg Environ Health. 10.1016/j.ijheh.2019.03.004 10.1016/j.ijheh.2019.03.004 30898527

[CR26] Toms LML, Braunig J, Vijayasarathy S, Phillips S, Hobson P, Aylward LL, Kirk MD, Mueller JF (2019b) Per-and polyfluoroalkyl substances (PFAS) in Australia: current levels and estimated population reference values for selected compounds. Int J Hyg Environ Health 222(3):387–394. 10.1016/j.ijheh.2019.03.004 30898527 10.1016/j.ijheh.2019.03.004

