# Supplementary material for: Identifying Populations with Elevated PFAS Exposure by Targeted Serum Sample Pooling
Source: Expo Health. 2025 May 20;17(4):1083–94. doi: 10.1007/s12403-025-00712-5 (PMC12254059; doi:10.1007/s12403-025-00712-5)
Supplement: Supplementary file 1 — Supplementary file1 (DOCX 219 KB) [file 12403_2025_712_MOESM1_ESM.docx]

**Identifying Populations with Elevated PFAS Exposure by Targeted Serum Sample Pooling**

Sandra Nilsson*^a^, Jennifer Bräunig^a,b^, Ava Mueller^a^, Nis-Julius Sontag^a^, Daman Langguth^c^, Carl Kennedy^c^, Peter Hobson^c^, Kevin V. Thomas^a^, Jochen F Mueller^a^, and Leisa-Maree Toms^d^

a. The University of Queensland, Queensland Alliance for Environmental Health Sciences, Woolloongabba, QLD, Australia

*b.* NSW Department of Climate Change, Energy, the Environment and Water, Environment Protection Science Branch, Lidcombe, NSW, Australia.

c. Sullivan Nicolaides Pathology, Bowen Hills, QLD, Australia

d. School of Public Health and Social Work, Faculty of Health, Queensland University of Technology, Kelvin Grove, QLD, Australia

Contents

[1. Analytical details and QAQC 3](#_Toc196384727)

[**Table S1.** Class, analyte, abbreviation, MRM transitions, internal standard, and method detection limits (MDL) 3](#_Toc196384728)

[**Table S2.** QA/QC outcomes. 4](#_Toc196384729)

[2. Additional Results Tables and Figures 5](#_Toc196384730)

[**Table S3**. Detection frequency and concentrations of PFAS in pooled serum from the Australian population collected in 2018-2019 and 2020-2021. 5](#_Toc196384731)

[**Table S4**. Concentrations of PFAS in pooled serum samples from PFAS hotspot Site 1. 7](#_Toc196384732)

[**Table S5**. Concentrations of PFAS in pooled serum samples from PFAS hotspot Site 2 and 3 8](#_Toc196384733)

[**Figure S1**. Age and sex trends of PFOA, PFHxS and PFOS concentrations in serum pools collected from the Australian population 9](#_Toc196384734)

[**Figure S2**. Supplementary to Figure 2 in the main manuscript.. 10](#_Toc196384735)

# Analytical details and QAQC

## **Table S1.** Class, analyte, abbreviation, MRM transitions, internal standard, and method detection limits (MDL)

|  |  |  |  | MDL (ng/mL) | |
| --- | --- | --- | --- | --- | --- |
| Analyte | Acronym | MRMs Q1/Q3 Mass(Da)* | Internal Standard | HBM 2018-19 | HBM 2020-21, Site 1-3 |
| Perfluoro-n-butanoic acid | PFBA | **212.8/169** | ^13^C_4_-PFBA | 0.13 | 0.38 |
| Perfluoro-n-pentanoic acid | PFPeA | **262.8/219** 262.8/69 | ^13^C_3_ PFPeA | 0.12 | 0.35 |
| Perfluoro-n-hexanoic acid | PFHxA | **312.8/269** 312.8/119 | ^13^C_2_-PFHxA | 0.07 | 0.20 |
| Perfluoro-n-heptanoic acid | PFHpA | **362.8/319** 362.8/169 | ^13^C_4_-PFHpA | 0.11 | 0.33 |
| Perfluoro-n-octanoic acid | PFOA | **412.8/369** 412.8/169 412.8/219 | ^13^C_4_-PFOA | 0.13 | 0.38 |
| Perfluoro-n-nonanoic acid | PFNA | **462.8/419** 462.8/169 | ^13^C_5_-PFNA | 0.14 | 0.42 |
| Perfluoro-n-decanoic acid | PFDA | **512.8/469** 512.8/269 | ^13^C_2_-PFDA | 0.14 | 0.43 |
| Perfluoro-n-undecanoic acid | PFUnDA | **562.8/519** 562.8/269 | ^13^C_2_PFUdA | 0.16 | 0.48 |
| Perfluoro-n-dodecanoic acid | PFDoDA | **612.8/569** 612.8/169 | ^13^C_2_PFDoA | 0.14 | 0.43 |
| Perfluoro-n-tridecanoic acid | PFTriDA | **662.8/619** 662.8/169 | ^13^C_2_PFDoA | 0.07 | 0.20 |
| Perfluoro-n-tetradecanoic acid | PFTreDA | **712.8/669** 712.8/169 | ^13^C_2_PFTeDA | 0.08 | 0.23 |
| Perfluoro-n-hexadecanoic acid | PFHxDA | **812.8/769** 812.8/169 | ^13^C_2_PFHxDA | 0.12 | 1.00 |
| Sodium perfluoro-1-propanesulfonate | PFPrS | **248.9/119.0** 248.9/80 | ^13^C_3_ PFBS | 1.00 | 1.00 |
| Potassium perfluoro-1-butanesulfonate | PFBS | **298.9/80** 298.9/99 | ^13^C_3_ PFBS | 0.11 | 0.33 |
| Sodium perfluoro-1-pentanesulfonate | PFPeS | **349/80** 349/99 | ^13^C_3_ PFBS | 0.09 | 0.28 |
| Sodium perfluoro-1-hexanesulfonate | PFHxS | **398.8/80** 398.8/99 398.8/119 398.8/130 | ^18^O_2_-PFHxS | 0.13 | 0.40 |
| Sodium perfluoro-1-heptanesulfonate | PFHpS | **448.8/80** 448.8/99 | ^18^O_2_-PFHxS | 0.13 | 0.40 |
| Sodium perfluoro-1-octanesulfonate | PFOS | 498.8/80 **498.8/99** 498.8/169 498.8/230 | ^13^C_4_-PFOS | 0.10 | 0.30 |
|  | Total PFOS ^2^ | 498.8/80 **498.8/99** 498.8/169 498.8/230 | ^13^C_4_-PFOS | 0.28 | 0.30 |
| Sodium perfluoro-1-nonanesulfonate | PFNS | **548.8/80** 548.8/99 | ^13^C_4_-PFOS | 0.13 | 0.38 |
| Sodium perfluoro-1-decanesulfonate | PFDS | **598.8/80** 598.8/99 | ^13^C_4_-PFOS | 0.13 | 0.40 |
| Sodium perfluoro-1-dodecanesulfonate | PFDoDS | **698.8/80** 698.8/99 | ^13^C_4_-PFOS | 0.49 | 1.48 |
| Perfluoro-1-butanesulfonamide | FBSA | **298.0 / 77.9** 298.0 / 63.9 | ^13^C_3_ PFBS | 1.00 | 1.00 |
| Perfluoro-1-hexanesulfonamide | FHxSA | **312.8 / 269.0** 312.8 / 119.0 | ^13^C_2_-PFHxA | 1.00 | 1.00 |
| Perfluoro-1-octanesulfonamide | FOSA | **497.8/78** 497.8/64 | ^13^C_8_-FOSA | 0.12 | 0.35 |
| N-methylperfluoro-1-octanesulfonamide | N-Me FOSA | **511.8/219** 511.8/169 | D_3_-N-Me FOSA | 0.16 | 0.48 |
| N,N-dimethylperfluoro-1-octanesulfonamide | N-Et FOSA | **525.8/169** 525.8/219 | D_5_-N-Et FOSA | 0.12 | 0.35 |
| Perfluorooctane sulfonamidoacetic acid | FOSAA |  |  | 0.15 | 0.45 |
| N-methylperfluoro-1-octanesulfonamidoacetic acid | N-Me FOSAA | **583.8/419** 583.8/219 | D_5_-N-Et FOSAA | 0.15 | 0.45 |
| N-ethylperfluoro-1-octanesulfonamidoacetic acid | N-Et FOSAA | **569.8/419** 569.8/512 | D_3_-N-Me FOSAA | 0.14 | 0.43 |
| 2-(N-methylperfluoro-1octanesulfonamido)-ethanol | N-Me FOSE | **630/59** | D_9_-N-Et FOSE | 0.08 | 0.23 |
| 2-(N-ethylperfluoro-1octanesulfonamido)-ethanol | N-Et FOSE | **616/59** | D_7_-N-Me FOSE | 0.13 | 0.40 |
| Sodium 1H,1H,2H,2H-perfluorohexane sulfonate (4:2) | 4:2 FTS | 326.8/307 **326.8/81** | ^13^C_2_ 4:2 FTS | 0.10 | 0.30 |
| Sodium 1H,1H,2H,2H-perfluorooctane sulfonate (6:2) | 6:2 FTS | 426.8/407 **426.8/81** | ^13^C_2_6:2 FTS | 0.20 | 0.60 |
| Sodium 1H,1H,2H,2H-perfluorodecane sulfonate (8:2) | 8:2 FTS | 526.8/507 **526.8/81** | ^13^C_2_-8:2 FTS | 0.14 | 0.43 |
| Sodium 1H,1H,2H,2H-perfluorododecane sulfonate (10:2) | 10:2 FTS | 626.9/607 **626.9/81** | ^13^C_2_-8:2 FTS | 0.07 | 0.28 |
| Perfluoroethylcyclohexane sulfonate | PFECHS | **460.9/381** 460.9/99 | ^13^C_4_-PFOS | 0.10 | 0.30 |
| Sodium 8-chloroperfluoro-1-octanesulfonate | 8Cl-PFOS | **514.8 / 99.0** 514.8 / 80.0 | ^13^C_4_-PFOS | 1.00 | 1.00 |
| 2,3,3,3-Tetrafluoro-2-(1,1,2,2,3,3,3-heptafluoropropoxyl)propanoic acid | GenX | **328.9 / 185.0** 328.9 / 119.0 | ^13^C_4_-PFOA | 1.00 | 1.00 |
| 2,3,3,3-Tetrafluoro-2-(1,1,2,2,3,3,3-heptafluoropropoxyl)propanoic acid | ADONA | **377.0 / 85.0** 377.0 / 251.0 | ^13^C_4_-PFOA | 1.00 | 1.00 |
| 2-perfluorodecyl ethanoic acid (10:2) | 10:2 FTCA | **577.0 / 63.0** 577.0 / 493.0 | ^13^C_2_PFDoA | 1.00 | 1.00 |
| Potassium 9-chlorohexadecafluoro-3-oxanonane-1-sulfonate | 9Cl-F53B | **530.7 / 350.9** 532.7 / 352.9 | ^13^C_4_-PFOS | 1.00 | 1.00 |
| Potassium 11-chloroeicosafluoro-3-oxaundecane-1-sulfonate | 11Cl-F53B | **630.7 / 450.8** 632.7 / 452.8 | ^13^C_4_-PFOS | 1.00 | 1.00 |
| Sodium bis(perfluorohexyl) phosphinate | 6:6 PFPiA | **700.9 / 400.7** 700.9 / 101.0 | ^13^C_2_PFDoA | 1.00 | 1.00 |

1. Transitions in bold are used for quantification
2. Total linear + branched isomers.

## **Table S2.** QA/QC outcomes.

|  | Duplicates,  CV (%) | | Replicates,  CV (%) |  | NIST 1957,  ng/mL | |  | External Quality Assessment Scheme* |  | Average Recovery Percent (%) | |
| --- | --- | --- | --- | --- | --- | --- | --- | --- | --- | --- | --- |
|  | Intra batch  (n=16 pairs) | Inter batch  (n=4 pairs) | Inter batch  (n=7) |  | Average (SD)  (n=16) | Reference values |  | Z score (Average) |  | Internal Standards | Native Standards |
| **PFBA** | 7% | 12% | 18% |  | <0.38 | n/a |  | n/a |  | 99 % | 103 % |
| **PFPeA** | n/a | n/a | n/a |  | <0.35 | n/a |  | n/a |  | 98 % | 106 % |
| **PFHxA** | n/a | n/a | n/a |  | <0.20 | n/a |  | 0.43 |  | 96 % | 104 % |
| **PFHpA** | 29% |  | 5% |  | 0.31 (0.12) | **0.31** |  | 0.24 |  | 94 % | 105 % |
| **PFOA** | 3% | 4% | 4% |  | 4.6 (0.28) | **5.00** |  | 0.11 |  | 107 % | 99 % |
| **PFNA** | 6% | 9% | 13% |  | 0.81 (0.07) | **0.88** |  | 1.89 |  | 92 % | 100 % |
| **PFDA** | 9% | 2% | 10% |  | 0.21 (0.07) | **0.39** |  | 0.13 |  | 107 % | 97 % |
| **PFUnDA** | n/a | n/a | 15% |  | 0.12 (0.03) | **0.17** |  | 0.80 |  | 107 % | 103 % |
| **PFDoDA** | n/a | n/a | n/a |  | <0.43 | n/a |  | n/a |  | 99 % | 100 % |
| **PFTriDA** | n/a | n/a | n/a |  | <0.2 | n/a |  | n/a |  | 87 % | 98 % |
| **PFTreDA** | n/a | n/a | n/a |  | <0.23 | n/a |  | n/a |  | 87 % | 97 % |
| **PFHxDA** | n/a | n/a | n/a |  | <1 | n/a |  | n/a |  | 54 % | 101 % |
| **PFPrS** | n/a | n/a | n/a |  | <1 | n/a |  | n/a |  | 101 % | 104 % |
| **PFBS** | n/a | n/a | n/a |  | <0.33 | n/a |  | n/a |  | 101 % | 106 % |
| **PFPeS** | n/a | n/a | n/a |  | <0.28 | n/a |  | n/a |  | 98 % | 103 % |
| **PFHxS** | 2% | 2% | 5% |  | 3.6 (0.19) | **4.00** |  | 0.53 |  | 96 % | 105 % |
| **PFHpS** | 3% | 14% | 6% |  | <0.4 | n/a |  | 0.18 |  | 96 % | 108 % |
| **PFOS** | 7% | 7% | 6% |  | 12 (0.57) | n/a |  | -0.01 |  | 98 % | 103 % |
| **Total PFOS^2^** | 5% | 2% | 4% |  | 19 (2.5) | **18.1** |  | -0.13 |  | 98 % | 105 % |
| **PFNS** | n/a | n/a | n/a |  | <0.38 | n/a |  | n/a |  | 98 % | 104 % |
| **PFDS** | n/a | n/a | n/a |  | <0.4 | n/a |  | n/a |  | 98 % | 101 % |
| **PFDoDS** | n/a | n/a | n/a |  | <1.48 | n/a |  | n/a |  | 99 % | 87 % |
| **FBSA** | n/a | n/a | n/a |  | <1 | n/a |  | n/a |  | 101 % | 101 % |
| **FHxSA** | n/a | n/a | n/a |  | <1 | n/a |  | n/a |  | 114 % | 102 % |
| **FOSA** | n/a | n/a | n/a |  | <0.35 | n/a |  | n/a |  | 114 % | 102 % |
| **N-Me FOSA** | n/a | n/a | n/a |  | <0.48 | n/a |  | n/a |  | 97 % | 93 % |
| **N-Et FOSA** | n/a | n/a | n/a |  | <0.35 | n/a |  | n/a |  | 83 % | 104 % |
| **FOSAA** | n/a | n/a | n/a |  | <0.45 | n/a |  | n/a |  | 134 % | 98 % |
| **N-Me FOSAA** | n/a | n/a | n/a |  | <0.45 | n/a |  | n/a |  | 134 % | 79 % |
| **N-Et FOSAA** | n/a | n/a | n/a |  | <0.43 | n/a |  | n/a |  | 152 % | 103 % |
| **N-Me FOSE** | n/a | n/a | n/a |  | <0.23 | n/a |  | n/a |  | 93 % | 104 % |
| **N-Et FOSE** | n/a | n/a | n/a |  | <0.4 | n/a |  | n/a |  | 79 % | 101 % |
| **4:2 FTS** | n/a | n/a | n/a |  | <0.3 | n/a |  | n/a |  | 110 % | 105 % |
| **6:2 FTS** | n/a | n/a | n/a |  | <0.6 | n/a |  | n/a |  | 157 % | 99 % |
| **8:2 FTS** | n/a | n/a | n/a |  | <0.43 | n/a |  | n/a |  | 162 % | 93 % |
| **10:2 FTS** | n/a | n/a | n/a |  | <0.28 | n/a |  | n/a |  | 99 % | 102 % |
| **PFECHS** | n/a | n/a | n/a |  | <0.3 | n/a |  | n/a |  | 98 % | 97 % |
| **8Cl-PFOS** | n/a | n/a | n/a |  | <1 | n/a |  | n/a |  | 98 % | 101 % |
| **GenX** | n/a | n/a | n/a |  | <1 | n/a |  | n/a |  | 107 % | 100 % |
| **ADONA** | n/a | n/a | n/a |  | <1 | n/a |  | n/a |  | 107 % | 101 % |
| **10:2 FTCA** | n/a | n/a | n/a |  | <1 | n/a |  | n/a |  | 99 % | 100 % |
| **9Cl-F53B** | n/a | n/a | n/a |  | <1 | n/a |  | n/a |  | 98 % | 102 % |
| **11Cl-F53B** | n/a | n/a | n/a |  | <1 | n/a |  | n/a |  | 98 % | 99 % |
| **6:6 PFPiA** | n/a | n/a | n/a |  | <1 | n/a |  | n/a |  | 99 % | 100 % |

1. External Quality Assessment Scheme; Centre de toxicologie-INSPQ. 2020 Performance
2. Total linear + branched isomers.

# Additional Results Tables and Figures

## **Table S3**. Detection frequency and concentrations of PFAS^1^(ng/mL) in pooled serum from the Australian population collected in 2018-2019 and 2020-2021 respectively^2^. The table shows results of PFAS detected in at least one sample.

|  |  |  | **2018-2019** | | | | |  |  | **2020-2021** | | | | |  |
| --- | --- | --- | --- | --- | --- | --- | --- | --- | --- | --- | --- | --- | --- | --- | --- |
|  |  |  | Males | |  | Females | |  |  | Males | |  | Females | |  |
| Analyte | Age group |  | >MDL (%) | Mean (SD)  ng/mL |  | >MDL (%) | Mean (SD)  ng/mL | Sex trend (P-value summary)^3^ |  | >MDL (%) | Mean (SD)  ng/mL |  | >MDL (%) | Mean (SD)  ng/mL | Sex trend (P-value summary)^3^ |
| PFBA |  |  |  |  |  |  |  |  |  |  |  |  |  |  |  |
|  | 0-4 |  | 100% | 0.22 (0.03) |  | 100% | 0.23 (0.02) | ns |  | 0% | <0.38 |  | 0% | <0.38 | n.a |
|  | 5-15 |  | 100% | 0.14 (0.01) |  | 100% | 0.16 (0.02) | ns |  | 0% | <0.38 |  | 0% | <0.38 | n.a |
|  | 16-30 |  | 75% | 0.14 (0) |  | 50% | 0.15 (0.02) | ns |  | 0% | <0.38 |  | 0% | <0.38 | n.a |
|  | 31-45 |  | 100% | 0.14 (0.01) |  | 100% | 0.15 (0.09) | ns |  | 0% | <0.38 |  | 0% | <0.38 | n.a |
|  | 46-60 |  | 0% | <0.13 |  | 25% | <0.13 | ns |  | 0% | <0.38 |  | 0% | <0.38 | n.a |
|  | >60 |  | 100% | 0.13 (0.01) |  | 100% | 0.14 (0.02) | ns |  | 0% | <0.38 |  | 0% | <0.38 | n.a |
| Age trend (P-value summary)^3^ | |  |  | ** |  |  | * |  |  |  | n.a |  |  | n.a |  |
| PFHpA |  |  |  |  |  |  |  |  |  |  |  |  |  |  |  |
|  | 0-4 |  | 100% | 0.22 (0.02) |  | 100% | 0.26 (0.04) | ns |  | 0% | <0.33 |  | 0% | <0.33 | n.a |
|  | 5-15 |  | 0% | <0.11 |  | 25% | <0.11 | n.a |  | 0% | <0.33 |  | 0% | <0.33 | n.a |
|  | 16-30 |  | 0% | <0.11 |  | 0% | <0.11 | n.a |  | 0% | <0.33 |  | 0% | <0.33 | n.a |
|  | 31-45 |  | 0% | <0.11 |  | 0% | <0.11 | n.a |  | 0% | <0.33 |  | 0% | <0.33 | n.a |
|  | 46-60 |  | 0% | <0.11 |  | 0% | <0.11 | n.a |  | 0% | <0.33 |  | 0% | <0.33 | n.a |
|  | >60 |  | 0% | <0.11 |  | 0% | <0.11 | n.a |  | 0% | <0.33 |  | 0% | <0.33 | n.a |
| Age trend (P-value summary)^3^ | |  |  | n.a |  |  | n.a |  |  |  | n.a |  |  | n.a |  |
| PFOA |  |  |  |  |  |  |  |  |  |  |  |  |  |  |  |
|  | 0-4 |  | 100% | 2.3 (0.24) |  | 100% | 2.7 (0.17) | * |  | 100% | 1.8 (0.04) |  | 100% | 1.8 (0.13) | ns |
|  | 5-15 |  | 100% | 1.8 (0.30) |  | 100% | 1.7 (0.12) | ns |  | 100% | 1.4 (0.16) |  | 100% | 1.3 (0.04) | ns |
|  | 16-30 |  | 100% | 1.6 (0.26) |  | 100% | 1.5 (0.24) | ns |  | 100% | 1.4 (0.20) |  | 100% | 1.4 (0.29) | ns |
|  | 31-45 |  | 100% | 1.9 (0.12) |  | 100% | 1.3 (0.11) | *** |  | 100% | 1.5 (0.16) |  | 100% | 1.2 (0.07) | * |
|  | 46-60 |  | 100% | 1.9 (0.13) |  | 100% | 1.7 (0.12) | ns |  | 100% | 1.8 (0.27) |  | 100% | 1.5 (0.15) | * |
|  | >60 |  | 100% | 2 (0.17) |  | 100% | 2.1 (0.23) | ns |  | 100% | 1.8 (0.10) |  | 100% | 2.0 (0.25) | ns |
| Age trend (P-value summary)^3^ | |  |  | ns |  |  | ns |  |  |  | ns |  |  | ns |  |
| PFNA |  |  |  |  |  |  |  |  |  |  |  |  |  |  |  |
|  | 0-4 |  | 100% | 0.46 (0.06) |  | 100% | 0.5 (0.06) | ns |  | 25% | <0.42 |  | 50% | <0.42 | n.a |
|  | 5-15 |  | 100% | 0.37 (0.05) |  | 100% | 0.37 (0.04) | ns |  | 25% | <0.42 |  | 50% | <0.42 | n.a |
|  | 16-30 |  | 100% | 0.33 (0.05) |  | 100% | 0.36 (0.07) | ns |  | 0% | <0.42 |  | 0% | <0.42 | n.a |
|  | 31-45 |  | 100% | 0.4 (0.02) |  | 100% | 0.35 (0.04) | ns |  | 0% | <0.42 |  | 0% | <0.42 | n.a |
|  | 46-60 |  | 100% | 0.4 (0.02) |  | 100% | 0.44 (0.05) | ns |  | 0% | <0.42 |  | 25% | <0.42 | n.a |
|  | >60 |  | 100% | 0.41 (0.03) |  | 100% | 0.47 (0.06) | ns |  | 25% | <0.42 |  | 100% | 0.50 (0.04) | n.a |
| Age trend (P-value summary)^3^ | |  |  | ns |  |  | ns |  |  |  | n.a |  |  | n.a |  |
| PFDA |  |  |  |  |  |  |  |  |  |  |  |  |  |  |  |
|  | 0-4 |  | 100% | 0.19 (0.02) |  | 100% | 0.22 (0.03) | ns |  | 0% | <0.43 |  | 0% | <0.43 | n.a |
|  | 5-15 |  | 100% | 0.25 (0.05) |  | 100% | 0.25 (0.03) | ns |  | 0% | <0.43 |  | 0% | <0.43 | n.a |
|  | 16-30 |  | 100% | 0.19 (0.03) |  | 75% | 0.23 (0.02) | ns |  | 0% | <0.43 |  | 0% | <0.43 | n.a |
|  | 31-45 |  | 100% | 0.2 (0.02) |  | 100% | 0.21 (0.03) | ns |  | 0% | <0.43 |  | 0% | <0.43 | n.a |
|  | 46-60 |  | 100% | 0.2 (0.01) |  | 100% | 0.23 (0.06) | ns |  | 0% | <0.43 |  | 0% | <0.43 | n.a |
|  | >60 |  | 100% | 0.33 (0.30) |  | 100% | 0.23 (0.03) | ns |  | 0% | <0.43 |  | 0% | <0.43 | n.a |
| Age trend (P-value summary)^3^ | |  |  | ns |  |  | ns |  |  |  | n.a |  |  | n.a |  |
| PFHxS |  |  |  |  |  |  |  |  |  |  |  |  |  |  |  |
|  | 0-4 |  | 100% | 1.1 (0.20) |  | 100% | 1.2 (0.11) | ns |  | 100% | 1.1 (0.26) |  | 100% | 1.2 (0.36) | ns |
|  | 5-15 |  | 100% | 1.6 (0.72) |  | 100% | 1.0 (0.21) | ns |  | 100% | 1.0 (0.36) |  | 100% | 0.80 (0.07) | ns |
|  | 16-30 |  | 100% | 2.1 (0.24) |  | 100% | 1.1 (0.25) | ** |  | 100% | 1.9 (0.38) |  | 100% | 1.0 (0.15) | ** |
|  | 31-45 |  | 100% | 2.6 (1.0) |  | 100% | 0.8 (0.05) | * |  | 100% | 2.1 (0.26) |  | 100% | 0.85 (0.04) | *** |
|  | 46-60 |  | 100% | 2.7 (0.96) |  | 100% | 1.4 (0.15) | * |  | 100% | 2.5 (0.64) |  | 100% | 1.2 (0.12) | ** |
|  | >60 |  | 100% | 2.2 (0.34) |  | 100% | 2.2 (0.33) | ns |  | 100% | 2.3 (0.29) |  | 100% | 2.2 (0.05) | ns |
| Age trend (P-value summary)^3^ | |  |  | ** |  |  | ** |  |  |  | *** |  |  | *** |  |
| PFHpS |  |  |  |  |  |  |  |  |  |  |  |  |  |  |  |
|  | 0-4 |  | 0% | <0.13 |  | 0% | <0.13 | n.a |  | 0% | <0.40 |  | 0% | <0.40 | n.a |
|  | 5-15 |  | 0% | <0.13 |  | 0% | <0.13 | n.a |  | 0% | <0.40 |  | 0% | <0.40 | n.a |
|  | 16-30 |  | 25% | <0.13 |  | 0% | <0.13 | n.a |  | 0% | <0.40 |  | 0% | <0.40 | n.a |
|  | 31-45 |  | 100% | 0.20 (0.02) |  | 0% | <0.13 | n.a |  | 0% | <0.40 |  | 0% | <0.40 | n.a |
|  | 46-60 |  | 100% | 0.29 (0.09) |  | 100% | 0.14 (0.03) | ** |  | 0% | <0.40 |  | 0% | <0.40 | n.a |
|  | >60 |  | 100% | 0.28 (0.02) |  | 100% | 0.23 (0.03) | ns |  | 0% | <0.40 |  | 0% | <0.40 | n.a |
| Age trend (P-value summary)^3^ | |  |  | n.a |  |  | n.a |  |  |  | n.a |  |  | n.a |  |
| PFOS |  |  |  |  |  |  |  |  |  |  |  |  |  |  |  |
|  | 0-4 |  | 100% | 1.5 (0.14) |  | 100% | 1.8 (0.28) | ns |  | 100% | 2.0 (1.21) |  | 100% | 1.5 (0.17) | ns |
|  | 5-15 |  | 100% | 2.1 (0.44) |  | 100% | 1.7 (0.17) | ns |  | 100% | 1.6 (0.13) |  | 100% | 1.5 (0.13) | ns |
|  | 16-30 |  | 100% | 2.7 (0.33) |  | 100% | 1.8 (0.18) | ** |  | 100% | 3.2 (1.31) |  | 100% | 1.7 (0.35) | *** |
|  | 31-45 |  | 100% | 3.9 (0.15) |  | 100% | 1.9 (0.25) | *** |  | 100% | 3.6 (0.35) |  | 100% | 2.0 (0.36) | *** |
|  | 46-60 |  | 100% | 6.0 (2.7) |  | 100% | 3.2 (0.54) | * |  | 100% | 4.5 (0.88) |  | 100% | 2.8 (0.70) | * |
|  | >60 |  | 100% | 5.3 (0.63) |  | 100% | 4.7 (0.64) | ns |  | 100% | 4.6 (0.38) |  | 100% | 4.3 (0.59) | ns |
| Age trend (P-value summary)^3^ | |  |  | *** |  |  | *** |  |  |  | *** |  |  | *** |  |
| Total PFOS^4^ | |  |  |  |  |  |  |  |  |  |  |  |  |  |  |
|  | 0-4 |  | 100% | 2.0 (0.14) |  | 100% | 2.4 (0.33) | ns |  | 100% | 3.1 (2.4) |  | 100% | 2.1 (0.13) | ns |
|  | 5-15 |  | 100% | 2.8 (0.59) |  | 100% | 2.3 (0.20) | ns |  | 100% | 2.2 (0.14) |  | 100% | 2.0 (0.15) | ns |
|  | 16-30 |  | 100% | 3.7 (0.37) |  | 100% | 2.5 (0.15) | ** |  | 100% | 4.3 (1.43) |  | 100% | 2.3 (0.3) | *** |
|  | 31-45 |  | 100% | 5.6 (0.38) |  | 100% | 2.6 (0.3) | *** |  | 100% | 4.8 (0.49) |  | 100% | 2.5 (0.42) | *** |
|  | 46-60 |  | 75% | 6.8 (0.74) |  | 100% | 4.3 (0.64) | ** |  | 100% | 6.3 (0.79) |  | 100% | 3.9 (0.83) | ** |
|  | >60 |  | 100% | 7.7 (0.74) |  | 100% | 6.9 (0.97) | ns |  | 100% | 6.7 (0.6) |  | 100% | 6.2 (0.70) | ns |
| Age trend (P-value summary)^3^ | |  |  | *** |  |  | *** |  |  |  | *** |  |  | *** |  |
| PFNS |  |  |  |  |  |  |  |  |  |  |  |  |  |  |  |
|  | 0-4 |  | 0% | <0.13 |  | 0% | <0.13 | n.a |  | 0% | <0.38 |  | 0% | <0.38 | n.a |
|  | 5-15 |  | 0% | <0.13 |  | 0% | <0.13 | n.a |  | 0% | <0.38 |  | 0% | <0.38 | n.a |
|  | 16-30 |  | 0% | <0.13 |  | 0% | <0.13 | n.a |  | 0% | <0.38 |  | 0% | <0.38 | n.a |
|  | 31-45 |  | 0% | <0.13 |  | 0% | <0.13 | n.a |  | 0% | <0.38 |  | 0% | <0.38 | n.a |
|  | 46-60 |  | 25% | <0.13 |  | 0% | <0.13 | n.a |  | 0% | <0.38 |  | 0% | <0.38 | n.a |
|  | >60 |  | 0% | <0.13 |  | 0% | <0.13 | n.a |  | 0% | <0.38 |  | 0% | <0.38 | n.a |
| Age trend (P-value summary)^3^ | |  |  | n.a |  |  | n.a |  |  |  | n.a |  |  | n.a |  |
| N-EtFOSE | |  |  |  |  |  |  |  |  |  |  |  |  |  |  |
|  | 0-4 |  | 0% | <0.13 |  | 0% | <0.13 | n.a |  | 0% | <0.40 |  | 0% | <0.40 | n.a |
|  | 5-15 |  | 0% | <0.13 |  | 0% | <0.13 | n.a |  | 0% | <0.40 |  | 0% | <0.40 | n.a |
|  | 16-30 |  | 0% | <0.13 |  | 0% | <0.13 | n.a |  | 0% | <0.40 |  | 0% | <0.40 | n.a |
|  | 31-45 |  | 0% | <0.13 |  | 0% | <0.13 | n.a |  | 0% | <0.40 |  | 0% | <0.40 | n.a |
|  | 46-60 |  | 0% | <0.13 |  | 0% | <0.13 | n.a |  | 100% | 0.60 (0.1) |  | 100% | 0.40 (0.10) | ns |
|  | >60 |  | 0% | <0.13 |  | 0% | <0.13 | n.a |  | 100% | 0.50 (0.17) |  | 100% | 0.50 (0.07) | ns |
| Age trend (P-value summary)^3^ | |  |  | n.a |  |  | n.a |  |  |  | n.a |  |  | n.a |  |

1. All PFAS are reported as the linear isomer concentration apart from ‘Total PFOS’ where the sum of both linear and branched isomers is reported (quantified by integrating both peaks)
2. Four pools for each age group and sex. Each pool consists of 100 individuals.
3. Age trend represent the correlation between average age and PFAS serum concentration (Pearson Correlation). For assessment of sex-trend, male and female pools were set as dichotomous variables (0/1 coding) (point-biserial Pearson Correlation). n.a.; not applicable (only PFAS with DF >75% were assessed), ns; not significant, *<0.05, **<0.01, ***:<0.001
4. Total linear + branched isomers.

## **Table S4**. Concentrations of PFAS^1^ in pooled serum samples from PFAS hotspot Site 1. Each stratum (age group and sex) consists of one pool. The table shows results of PFAS detected in at least one sample.

| Site 1 |  |  |  | |
| --- | --- | --- | --- | --- |
| PFAS (ng/mL serum) | Age-group | No of individuals in each pool  Males/Females | Males | Females |
| **PFOA** |  |  | ng/mL | |
|  | 5-15 | 15 | 1.1 | |
|  | 16-30 | 88/145 | 1.8 | 1.2 |
|  | 31-45 | 62/98 | 1.4 | 0.98 |
|  | 46-60 | 45/70 | 1.5 | 1.4 |
|  | >60 | 25/35 | 1.7 | 2.1 |
| **PFNA** |  |  |  |  |
|  | 5-15 | 15 | <0.42 | |
|  | 16-30 | 88/145 | 0.48 | <0.42 |
|  | 31-45 | 62/98 | <0.42 | <0.42 |
|  | 46-60 | 45/70 | <0.42 | <0.42 |
|  | >60 | 25/35 | <0.42 | 0.502 |
| **PFDA** |  |  |  |  |
|  | 5-15 | 15 | <0.43 | |
|  | 16-30 | 88/145 | <0.43 | <0.43 |
|  | 31-45 | 62/98 | <0.43 | <0.43 |
|  | 46-60 | 45/70 | <0.43 | 0.44 |
|  | >60 | 25/35 | <0.43 | 1.645 |
| **PFHxS** |  |  |  |  |
|  | 5-15 | 15 | 2.2 | |
|  | 16-30 | 88/145 | 4.1 | 1.9 |
|  | 31-45 | 62/98 | 5.0 | 2.2 |
|  | 46-60 | 45/70 | 4.2 | 3.8 |
|  | >60 | 25/35 | 4.7 | 4.0 |
| **PFOS** |  |  |  |  |
|  | 5-15 | 15 | 1.3 | |
|  | 16-30 | 88/145 | 3.5 | 1.7 |
|  | 31-45 | 62/98 | 5.5 | 2.2 |
|  | 46-60 | 45/70 | 3.1 | 3.1 |
|  | >60 | 25/35 | 4.2 | 2.9 |
| **Total PFOS** |  |  |  |  |
|  | 5-15 | 15 | 2.3 | |
|  | 16-30 | 88/145 | 5.7 | 2.9 |
|  | 31-45 | 62/98 | 7.5 | 3.2 |
|  | 46-60 | 45/70 | 5.4 | 5.1 |
|  | >60 | 25/35 | 7.2 | 5.3 |

1. All PFAS are reported as the linear isomer concentration apart from ‘Total PFOS’ where the sum of both linear and branched isomers is reported (quantified by integrating both peaks)

## **Table S5**. Concentrations of PFAS^1^(ng/mL) in pooled serum samples from PFAS hotspot Site 2 and 3. Each stratum (radius from source and sex) consists of multiple pools of 10 individuals (all ages) in each pool. The table shows results of PFAS detected in at least one sample.

|  |  |  | **Site 2** | | | | |  | **Site 3** | | | | |
| --- | --- | --- | --- | --- | --- | --- | --- | --- | --- | --- | --- | --- | --- |
|  |  |  | Males | |  | Females | |  | Males | |  | Females | |
| PFAS (ng/mL serum) | Radius from source |  | >MDL (%) | Mean (SD)  ng/mL |  | Mean (SD)  ng/mL | Mean (SD) |  | >MDL (%) | Mean (SD)  ng/mL |  | >MDL (%) | Mean (SD)  ng/mL |
| **PFHpA** |  |  |  |  |  |  |  |  |  |  |  |  |  |
|  | 5km |  | 5% | <0.33 |  | 0% | <0.33 |  | 0% | <0.33 |  | 22% | <0.33 |
|  | 10km |  | 0% | <0.33 |  | 4% | <0.33 |  | 0% | <0.33 |  | 0% | <0.33 |
| **PFOA** |  |  |  |  |  |  |  |  |  |  |  |  |  |
|  | 5km |  | 100% | 1.7 (0.31) |  | 100% | 1.6 (0.42) |  | 100% | 2.3 (0.54) |  | 100% | 2.0 (0.48) |
|  | 10km |  | 100% | 1.8 (0.3) |  | 100% | 1.5 (0.38) |  | 100% | 2.2 (0.39) |  | 100% | 1.8 (0.41) |
| **PFNA** |  |  |  |  |  |  |  |  |  |  |  |  |  |
|  | 5km |  | 40% | <0.42 |  | 41% | <0.42 |  | 57% | 0.5 (0.2) |  | 89% | 0.53 (0.18) |
|  | 10km |  | 52% | <0.42 |  | 40% | <0.42 |  | 67% | 0.45 (0.12) |  | 64% | 0.44 (0.14) |
| **PFDA** |  |  |  |  |  |  |  |  |  |  |  |  |  |
|  | 5km |  | 0% | <0.43 |  | 0% | <0.43 |  | 14% | <0.43 |  | 22% | <0.43 |
|  | 10km |  | 0% | <0.43 |  | 0% | <0.43 |  | 11% | <0.43 |  | 7% | <0.43 |
| **PFHxS** |  |  |  |  |  |  |  |  |  |  |  |  |  |
|  | 5km |  | 100% | 1.9 (0.69) |  | 100% | 1.2 (0.45) |  | 100% | 3.7 (0.75) |  | 100% | 2.2 (0.63) |
|  | 10km |  | 100% | 2.4 (1.1) |  | 100% | 1.2 (0.53) |  | 100% | 3 (0.41) |  | 100% | 2 (0.54) |
| **PFHpS** |  |  |  |  |  |  |  |  |  |  |  |  |  |
|  | 5km |  | 0% | <0.40 |  | 0% | <0.40 |  | 14% | <0.40 |  | 0% | <0.40 |
|  | 10km |  | 0% | <0.40 |  | 0% | <0.40 |  | 0% | <0.40 |  | 0% | <0.40 |
| **PFOS** |  |  |  |  |  |  |  |  |  |  |  |  |  |
|  | 5km |  | 100% | 3.5 (1.1) |  | 100% | 2.4 (1.03) |  | 100% | 6.9 (4.8) |  | 100% | 3.4 (0.9) |
|  | 10km |  | 100% | 3.7 (1) |  | 100% | 2.7 (1) |  | 100% | 4.7 (1.1) |  | 100% | 4.1 (2.4) |
| **Total PFOS** |  |  |  |  |  |  |  |  |  |  |  |  |  |
|  | 5km |  | 100% | 5.7 (2.6) |  | 100% | 3.8 (1.8) |  | 100% | 8.9 (5.2) |  | 100% | 4.7 (1.2) |
|  | 10km |  | 100% | 5.2 (1.2) |  | 100% | 4 (1.5) |  | 100% | 6.9 (1.5) |  | 100% | 5.3 (2.5) |
| **N-Me FOSE** |  |  |  |  |  |  |  |  |  |  |  |  |  |
|  | 5km |  | 5% | <0.23 |  | 0% | <0.23 |  | 0% | <0.23 |  | 11% | <0.23 |
|  | 10km |  | 0% | <0.23 |  | 8% | <0.23 |  | 0% | <0.23 |  | 0% | <0.23 |
| **PFECHS** |  |  |  |  |  |  |  |  |  |  |  |  |  |
|  | 5km |  | 0% | <0.30 |  | 0% | <0.30 |  | 0% | <0.30 |  | 22% | <0.30 |
|  | 10km |  | 0% | <0.30 |  | 0% | <0.30 |  | 0% | <0.30 |  | 0% | <0.30 |

1. All PFAS are reported as the linear isomer concentration apart from ‘Total PFOS’ where the sum of both linear and branched isomers is reported (quantified by integrating both peaks)

## **Figure S1**. Age and sex trends of PFOA, PFHxS and PFOS concentrations(ng/mL) in serum pools collected from the Australian population (Australian HBM project) in 2018-2019 and 2020-2021 as part of the current study.

## **Figure S2**. Supplementary to Figure 2 in the main manuscript. In figure two, one outlier sample from the Australian HBM project was excluded (one pool among males). In the current comparison, all pools are included. Concentrations (ng/mL) of PFOA, PFHxS and PFOS in pools collected from site 2 and 3 in 2019, and how they compare to the mean Australian HBM project pools (blue) in 2018-2019. Pools collected as part of the Australian HBM project were made up from 100 individuals/pool, where 24 pools were collected for each sex. Pools collected from sites 2 and 3 were made up from 10 individuals/pools, where 20-25 pools were collected from each stratum at site 2, and 7-14 pools were collected from each stratum at site 3.
